# Burrow systems evince non-solitary geomyid rodents from the Paleogene of southern Mexico

**DOI:** 10.1371/journal.pone.0230040

**Published:** 2020-03-12

**Authors:** Rosalía Guerrero-Arenas, Eduardo Jiménez-Hidalgo, Jorge Fernando Genise

**Affiliations:** 1 Laboratorio de Paleobiología, Universidad del Mar, Oaxaca, México; 2 CONICET, División Icnología, Museo Argentino de Ciencias Naturales, Buenos Aires, Argentina; Mansoura University, EGYPT

## Abstract

We describe a new complex burrow system produced by geomyids in southern Mexico. *Yaviichnus inyooensis* igen. isp. nov. is composed of main large chambers near the top of the paleosol, from which shafts showing different morphologies and orientations radiate, some of them ending in or connected to small deeper chambers. *Gregorymys* spp. is proposed as the producer based on its fossorial habits, abundance in the outcrops, presence of remains inside the burrows, and paired grooves in the walls, which are compatible with the traces of geomyid incisors. The complexity of these burrows attests to an extended underground life that would have been triggered by semiarid to arid conditions. Morphological complexity also suggests that the burrows were excavated and inhabited by more than one individual, indicating that Oligocene *Gregorymys* of southern Mexico would be a unique gregarious geomyid.

## Introduction

The behavior of burrowing has probably been present in mammals since their early origins. Soil provides physical protection; it also supports plants and animals that many fossorial mammals use [1]. Underground shelter provides two main services: protection from predators and from environmental fluctuations or extreme conditions predominating above the ground [2].

It is assumed that subterranean mammals exploited the underground ecotope during the global climatic transition from the middle Eocene to the early Oligocene [3]. There are several early Oligocene localities in temperate North America, but the only reported Oligocene mammalian locality from tropical North America is Santiago Yolomecatl in southern Mexico [4]. It includes a few fossorial taxa, such as *Rhineura* (Reptilia: Squamata), and rodents (*Gregorymys veloxikua*, *Gregorymys* sp. and Florentiamyidae indet.) [5,6]. Several specimens of *G*. *veloxikua* and *G*. sp. had been collected inside burrows, which were tentatively identified as *Alezichnos* isp. [6]; however, further detailed study on these burrows suggested that these structures were much more complex than *Alezichnos*.

The objectives of this research are: (1) to describe the new complex burrow system and to include it in an ichnotaxonomical frame; (2) to test the hypothesis that a species of *Gregorymys* was the system’s producer; (3) to discuss the factors that promoted the development of such complex burrow systems; and (4) to analyze the possibilities that gregarious geomyids were present during the Oligocene in southern Mexico.

### Geological setting

**Fig 1:**
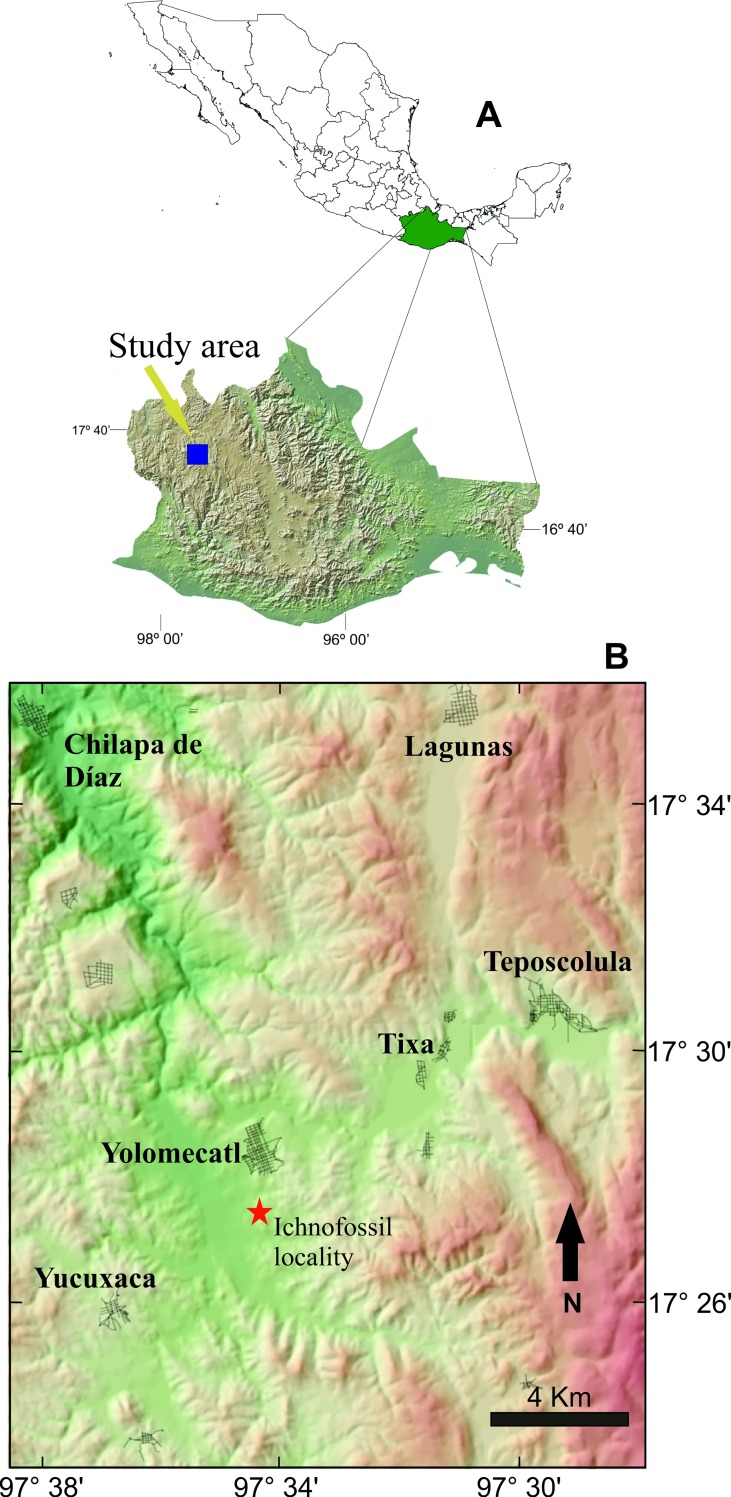
Study area in southern Mexico.

**Fig 2:**
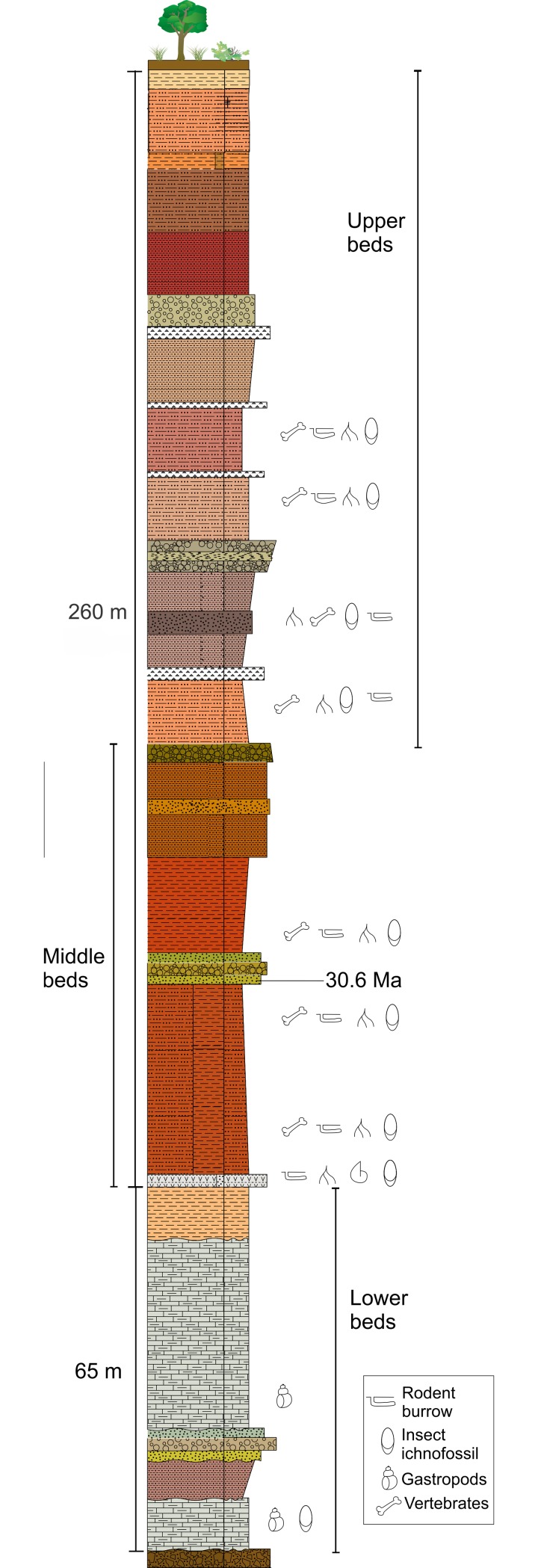
Generalized stratigraphic column of the oligocene sequence of Santiago Yolomecatl, Oaxaca, southern Mexico.

The study area is in northwestern Oaxaca state, in southern Mexico (Fig 1). Fossiliferous outcrops are within the municipality of Santiago Yolomecatl. Lithological units represent a fluviolacustrine succession with several paleosol horizons (Fig 2). Stratigraphical description of the study zone had previously been reported in detail [5–7].

**Fig 3:**
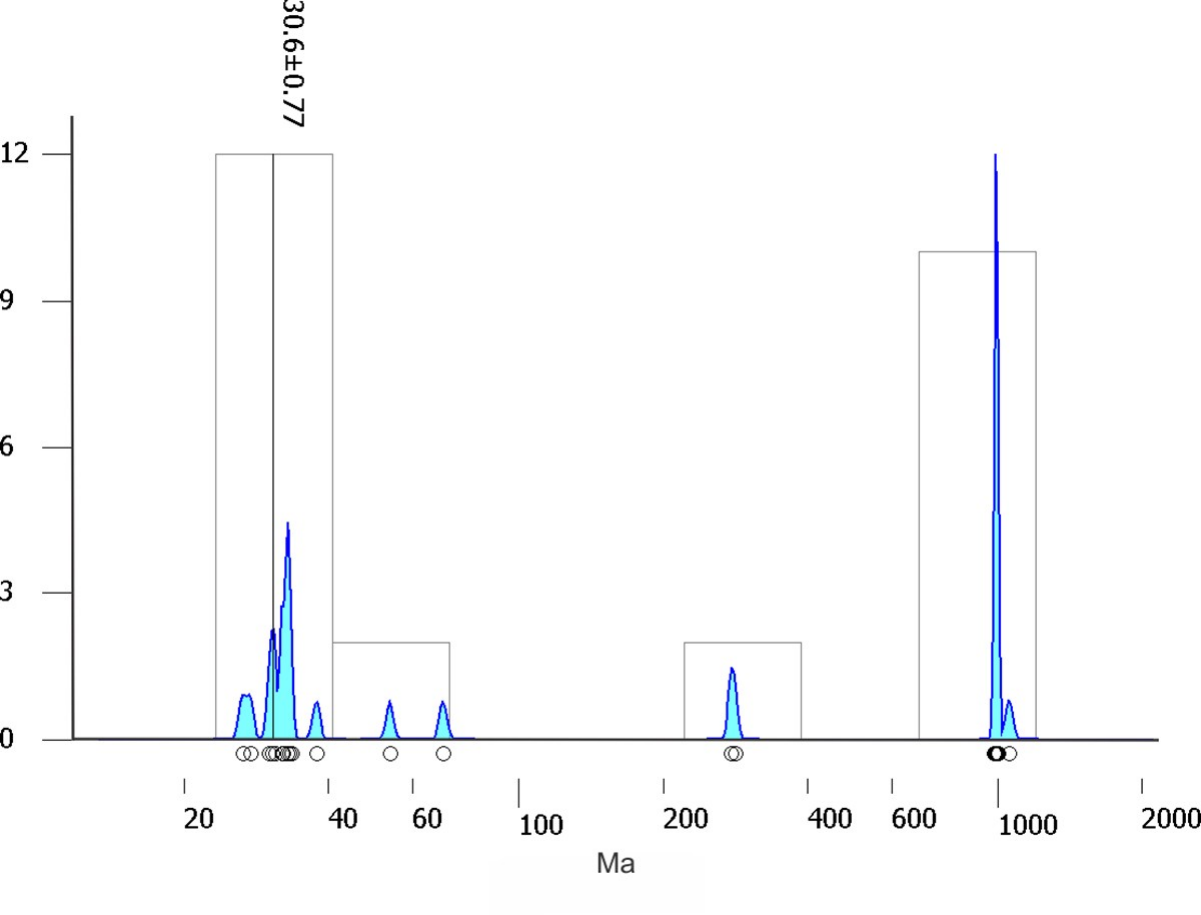
Maximum depositional age biased on U-Pb detrital zircon geochronology.

The fossiliferous deposit was originally regarded as late Eocene given the presence of *Miohippus assiniboiensis* and previously published radiometric dates from overlying andesites outside of the study area [5]. Before it was known that the Yolomecatl sequence was fossiliferous, it was considered part of the late Eocene–early Oligocene Chilapa Formation [8]. Years later, it was considered a new geologic formation of middle Eocene age, based on a ^40^Ar-^39^Ar age of 40.3 ± 1.0 Ma [9]. We could not locate the dated tuff at the reported location (see table B.4 of [9]), so to obtain a more precise age estimation of the fossiliferous beds of Yolomecatl, U-Pb detrital zircon geochronology was used to determine the maximum depositional age of a conglomeratic sandstone bed that is within the fossiliferous beds (Fig 2) (S1 File). Its maximum depositional age was estimated at 30.6 Ma (Fig 3), placing the age of the Yolomecatl deposits, and their fossils (Iniyoo Local Fauna) in the early Oligocene. Some newly collected mammalian taxa (*Oreodontoides*, *Mammacyon*, *Cormocyon*) also indicate an early Oligocene age (Arikareean 1 North American Land Mammal Age) from their sedimentary sequence [4]. This new age agrees with the previously reported age of deposition for the Chilapa Formation, which was considered to be 35.6 to 29 Ma [8]. Additionally, new regional stratigraphic relationships, as well as petrographic and mineralogical data indicate that the fossiliferous beds of Yolomecatl represent the marginal facies of the Chilapa Formation [10].

Strata from the Yolomecatl succession can be informally subdivided in three sections (Fig 2): the “lower beds” are a sequence of limestone of freshwater origin and shale strata, with siltstone, sandstone and conglomerate intercalations; the “middle beds” consist of a sequence of clayey silt and silty sandstone with sandstone and conglomerate interbedding; the “upper beds” are a diverse sequence consisting of clayey siltstone, silty sandstone, silcretes, sandstone and conglomerate strata. Fossils and trace fossils are present along the whole sequence; vertebrate burrows appear in the “lower beds” where they are isolated and scarce. The burrows are particularly abundant in some levels of the “middle beds” where they compose complex systems or are isolated. Burrow abundance decreases in the “upper beds” where only a few isolated specimens occur. Vertebrate burrows occur in paleosols developed in floodplains and lake shores [10].

Most of the large casts of chambers and tunnels are preserved in full relief in the field. Systems cover approximately 100 m^2^ in the best-preserved exposures. Since the systems crop out in ravines and uncovered soils, weathering by several agents (wind, water and cattle) is a permanent menace for the trace fossils’ preservation.

### Nomenclatural acts

The electronic edition of this article conforms to the requirements of the amended International Code of Zoological Nomenclature, and hence the new names contained herein are available under that Code from the electronic edition of this article. This published work and the nomenclatural acts it contains have been registered in ZooBank, the online registration system for the ICZN. The ZooBank LSIDs (Life Science Identifiers) can be resolved and the associated information viewed through any standard web browser by appending the LSID to the prefix "http://zoobank.org/". The LSID for this publication is: urn:lsid:zoobank.org:pub: F91D79B9-A430-40B4-8493-46970B077EFB. The electronic edition of this work was published in a journal with an ISSN, and has been archived and is available from the following digital repositories: PubMed Central and LOCKSS.

## Materials and methods

Diagnosis, descriptions and surface morphology of walls were based on the best-exposed or -preserved specimens. Chamber shape may be more deformed by carbonate cementation in some stratigraphic levels than in others, so we only included measurements of chambers from the “middle beds”, which preserve the best examples. There is no evidence of sediment compaction in the burrowing fossiliferous levels. When the burrows were accessible, measurements were taken in the field. When they were located in inaccessible vertical exposures, digital photographs and Image-J software were used for measurements.

Holotype and all collected specimens for this study are deposited in the Colección de Icnología, Laboratorio de Paleobiología, Laboratorio de Paleobiología, campus Puerto Escondido, Universidad del Mar. Collection address is Km. 2.5 Carretera Sola de Vega-Puerto Escondido, San Pedro Mixtepec. C.P. 71980 Oaxaca, México. Since the fossil collection is into a public educational institution, it is accessible to the all the interested researchers, via authorization by the Collection Manager.

All the paleoichnological specimens are deposited in this collection under the acronym UMPLIC-. Types were selected among the relatively short pieces of tunnels that were collected because it is impossible to collect entire large burrow systems. They were based on the preservation quality of bioglyphs and completeness. Specimens used in the study are UMPLIC-377 to -388, -390, -392, -395, and -403.

The permits of prospecting the fossiliferous localities were conferred by main local authorities of Santiago Yolomécatl: Rogelio Martínez Ramírez (*Presidente Municipal*, Municipal President), and Martha Karla Cervantes Ramírez, (*Síndica municipal*, a kind of Deputy Mayor). They approved them in a formal presentation in the beginning of the fieldtrip season.

Specific permits for collecting are not mandatory, because the Mexican legislation currently does not require them. However, in order to have all the possible regulations, all specimens were registered in the Dirección de Registro Público de Monumentos y Zonas Arqueológicos e Históricos of Instituto Nacional de Antropología e Historia (INAH). Universidad del Mar is registered as the legal custodian of the specimens in Dirección de Registro Público de Monumentos y Zonas Arqueológicos e Históricos of INAH, the national database of paleontological monuments of the Mexican instance in charge of the preservation and custody of Mexican fossils. All the burrows are registered in this database. Registry number of Universidad del Mar (as a legal custodian) in this database is 3024 P.M.

## Results

### Systematic paleontology

***Yaviichnus*** igen nov urn: urn:lsid:zoobank.org:act:89F41D59-0510-4079-BC4C-415A4D40AED6

**Etymology.** Derived from *Yavi*, from the Mixteco language (typical of the region of Oaxaca where the study zone is placed), meaning “rodent cave”; *ichnus* is derived from the Greek *Ikhnos*, meaning “trace”.

**Fig 4:**
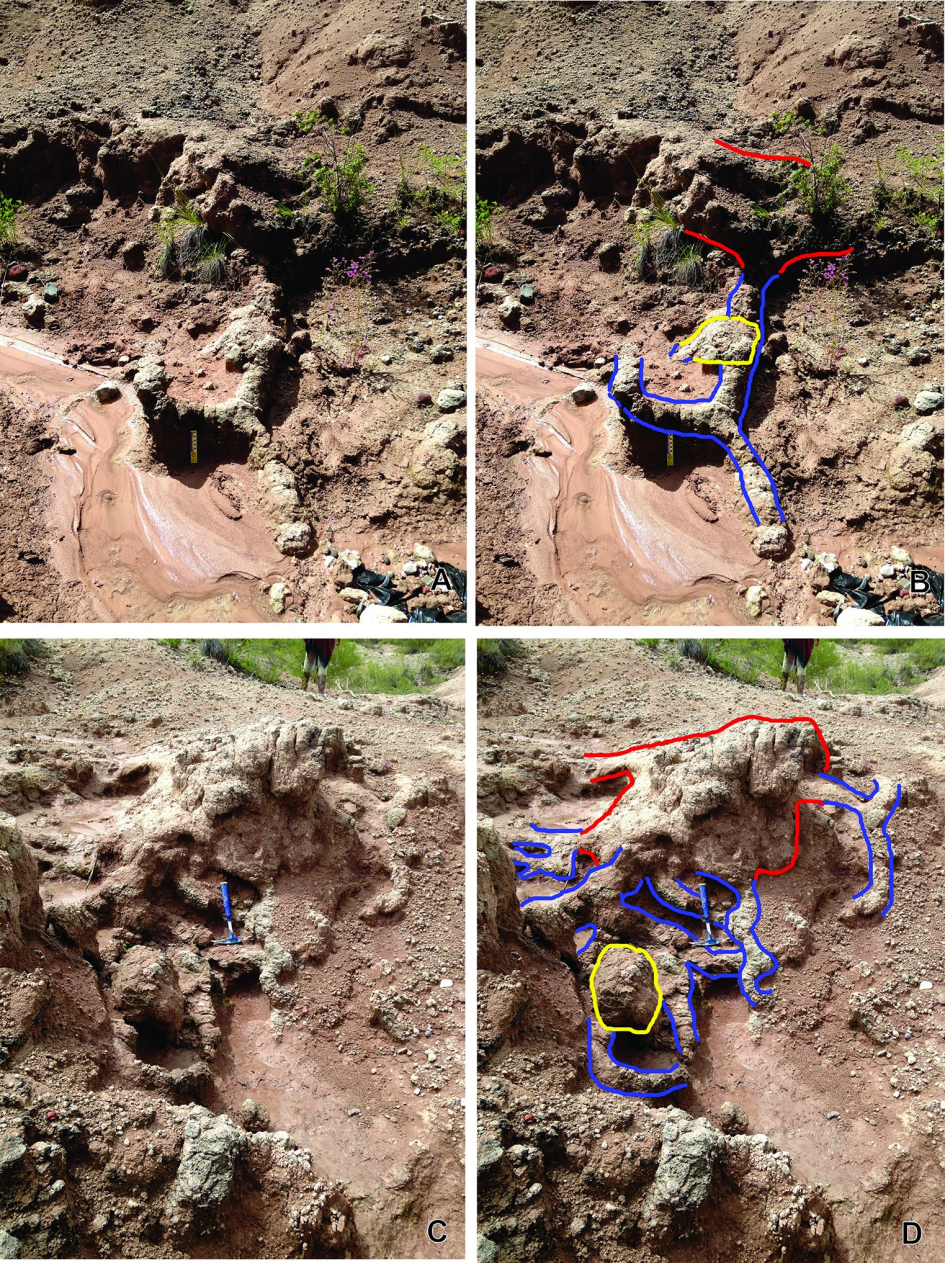
*Yaviichnus iniyooensis* morphology. (A, C) Systems composed by a main chamber (red delineated in B, D), secondary chamber (yellow delineated in B, D) and tunnels (blue delineated in B, D). (A, C) Burrow systems are *in situ*, in “middle beds” strata.

**Fig 5:**
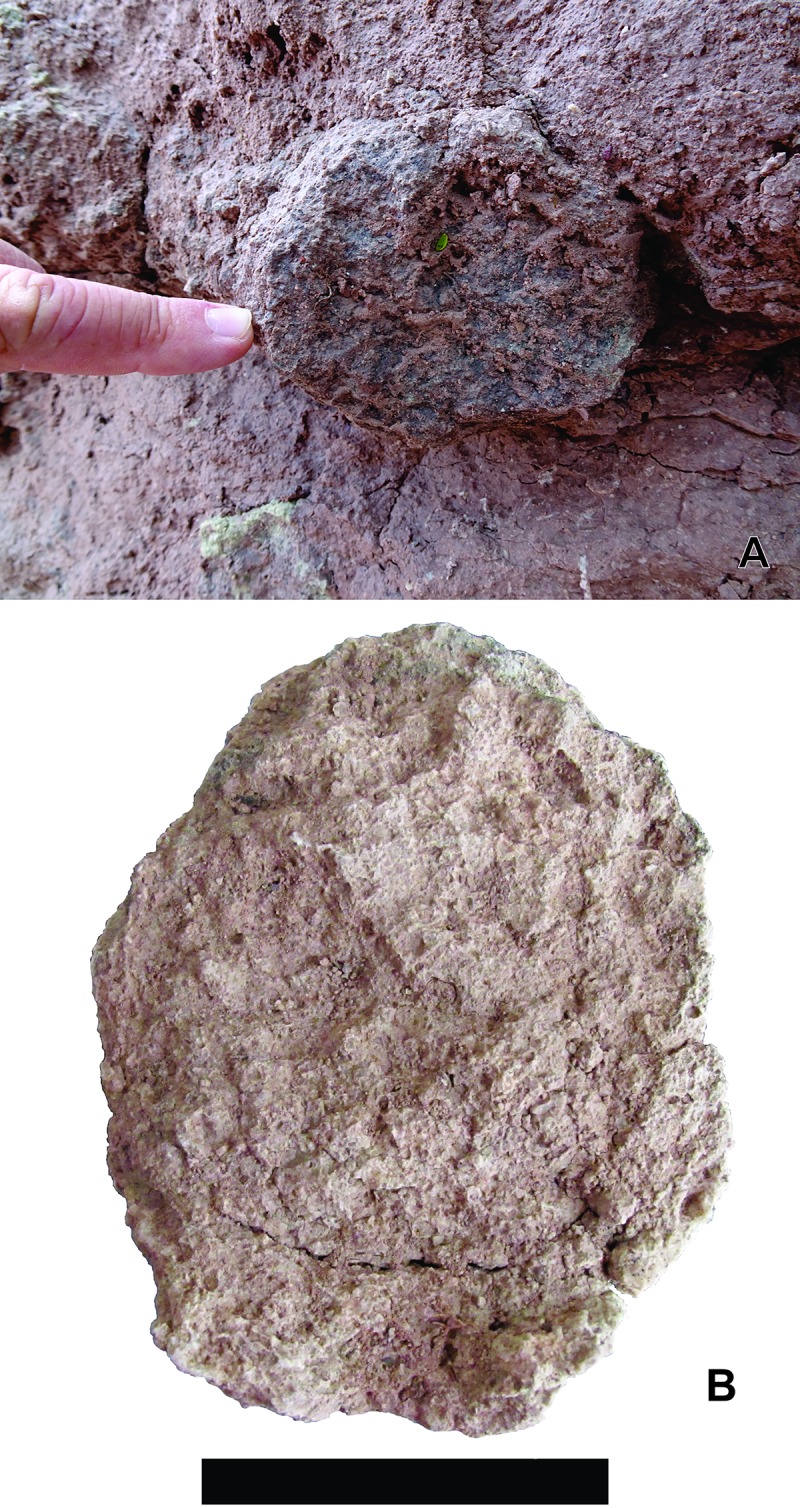
*Yaviichnus iniyooensis* cross-section of tunnels. (A) Horizontal tunnel cross-section *in situ*. From “middle beds” strata. (B) Paratype UMPLIC- 386, elliptical cross-section of a tunnel. Scale = 7 mm.

**Fig 6:**
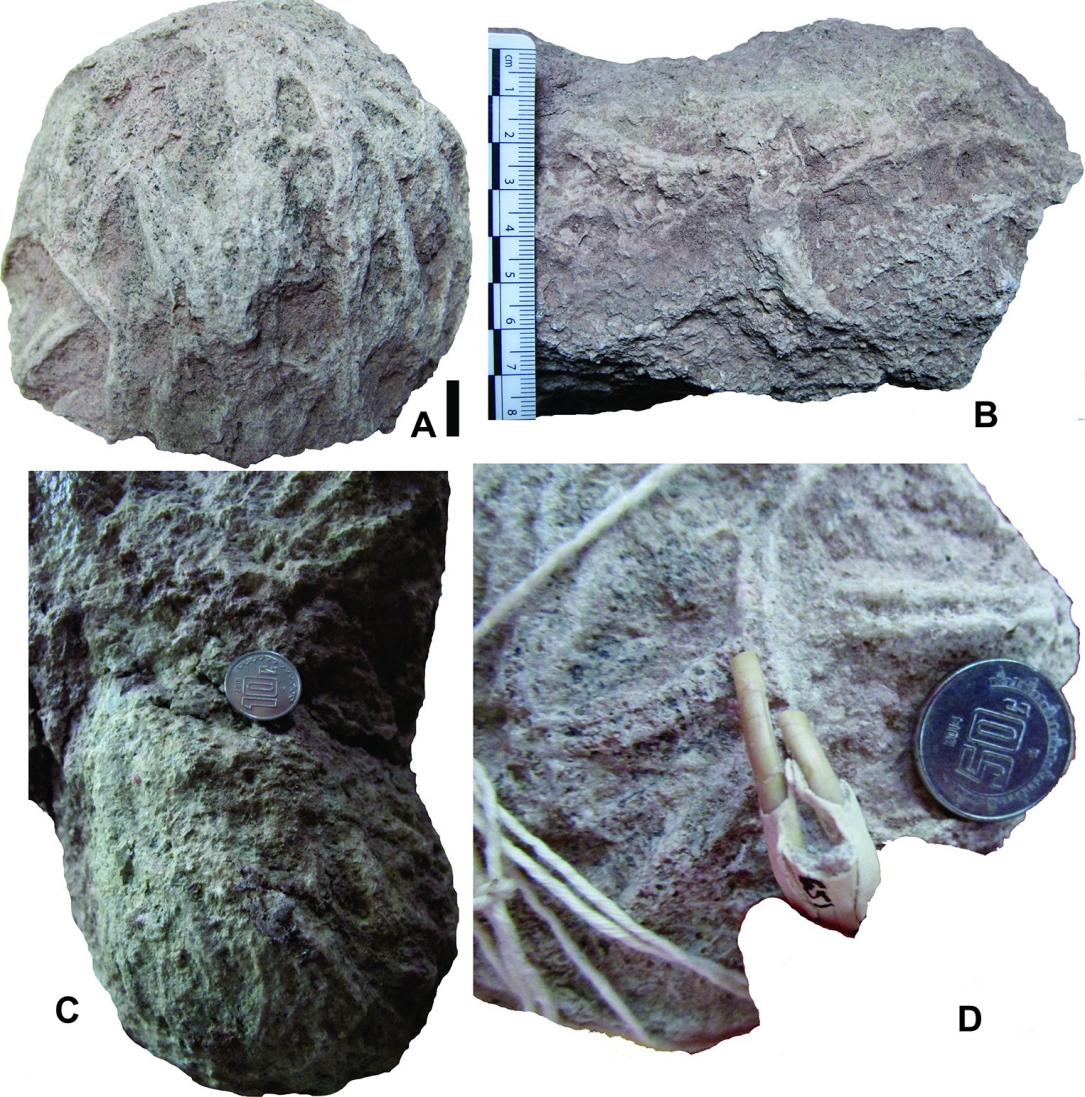
*Yaviichnus iniyooensis* bioglyphs. (A) Paratype UMPLIC-378, end of tunnel, covered with incisor marks. Scale = 1.3 mm. (B) Paratype UMPLIC-381, incisor marks on the cast surface of a tunnel segment. (C) Paratype UMPLIC-403, incisor marks on the cast surface of the end tunnel. Coin diameter = 14 mm. (D) Comparison of the marks, paratype UMPLIC-378 with incisor width of *G*. *veloxikua* specimen UMPE-671. Coin diameter = 16 mm.

**Diagnosis.** Interconnected burrow system composed of shafts, tunnels and two types of chambers (Fig 4). Large- to medium-sized superior chambers are connected to descending, radiating and inclined shafts, or to horizontal tunnels. Smaller secondary chambers are present at the end of these burrows or lateral to them (Fig 4). Horizontal burrows are straight, sinuous or show “C” or “H” paths (Fig 4). Vertical to sub-vertical burrows are straight, curved, sinuous or show consecutive arches resembling roughly a helical design (Fig 4). Tunnels and shafts are branched or simple. Horizontal burrows are wider than they are tall (Fig 5A), whereas vertical ones are almost circular in cross section. Surface morphology of some burrows includes short, straight, paired marks on the external surface of the burrow fill (Fig 6A–6C).

**Remarks.** No reported ichnotaxon shows this unique combination of tunnels and chambers characteristics, burrow system architecture and bioglyph characteristics.

### *Yaviichnus iniyooensis* isp. nov.

**Etymology.** After *Iniyoo*, derived from the Mixteco language, meaning “two hearts.” *Iniyoo* is the Mixteco name of Yolomecatl, where the fossiliferous strata crop out.

**Holotype.** UMPLIC-392, a fragment of a burrow fill from the “upper beds” of the Yolomecatl strata of the Chilapa Formation.

**Occurrence.** Only known from the early Oligocene beds (Arikareean 1 North American Land Mammal Age) of the Yolomecatl succession of the Chilapa Formation.

**Examined material.** More than 100 casts were observed and examined in the field. Three types of internal casts were collected to detail descriptions: a) End of tunnels: UMPLIC-378 (Fig 6A) and UMPLIC-390 from the “upper beds”; UMPLIC-379 and UMPLIC-403 from the “middle beds”; b) Fragments of three-branched tunnel: UMPLIC-382 from the “middle beds”; c) Fragmented burrows showing well preserved bioglyphs on the surface: UMPLIC-381 (Fig 6B) and UMPLIC-386 from the “upper beds”; UMPLIC-380 (Fig 6A) and UMPLIC-383 from the “middle beds.” All of them are from the Yolomecatl strata of the Chilapa Formation.

**Diagnosis.** Only known ichnospecies, same as for ichnogenus.

**Fig 7:**
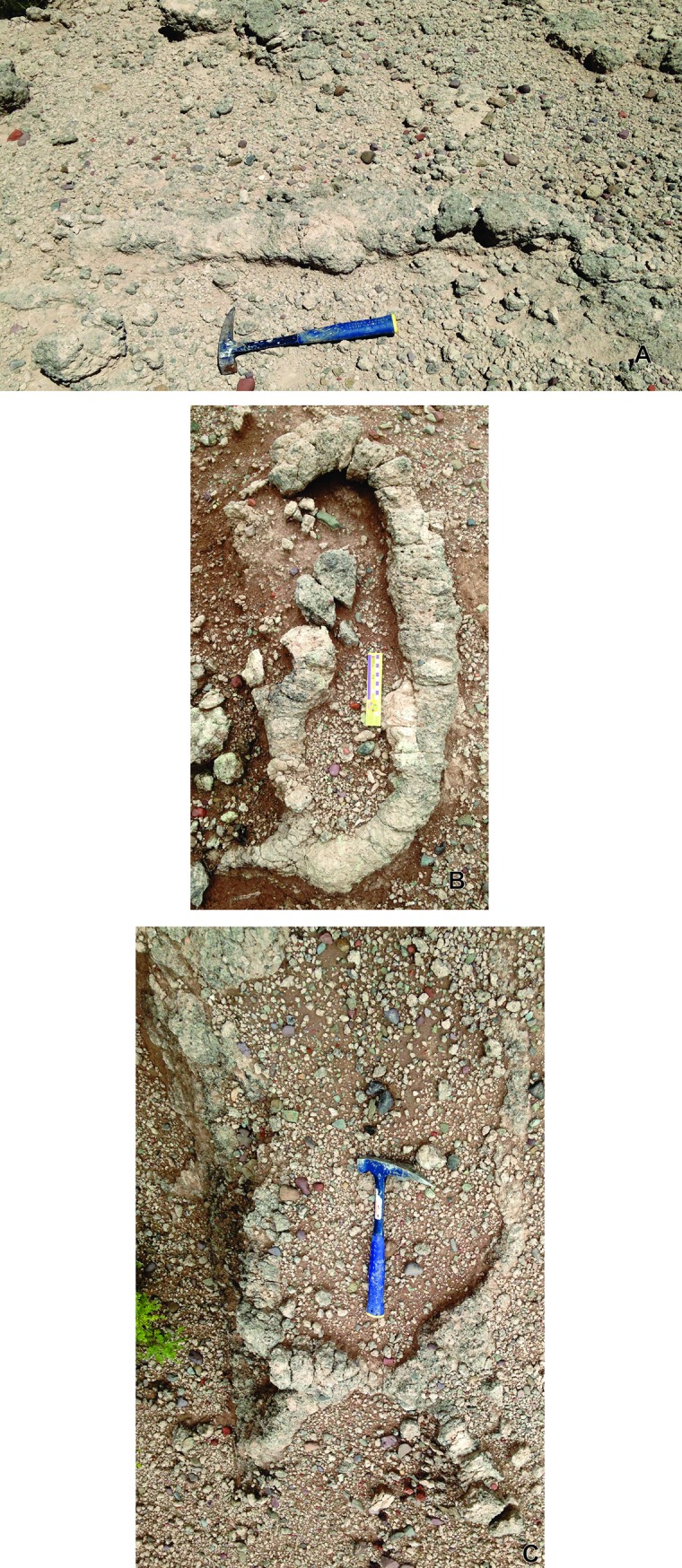
General morphology of *Y*. *iniyooensis* horizontal to sub-horizontal tunnels. (A) Horizontal, straight segment tunnel. Scale = 34.3 cm. (B) “C” shape of a segment tunnel. Divisions of the rule are in centimeters; total length of the rule = 17 cm. (C) “H” shape of a segment tunnel. Scale = 34.3 cm. All the burrows are *in situ*, in “middle beds” strata.

**Fig 8:**
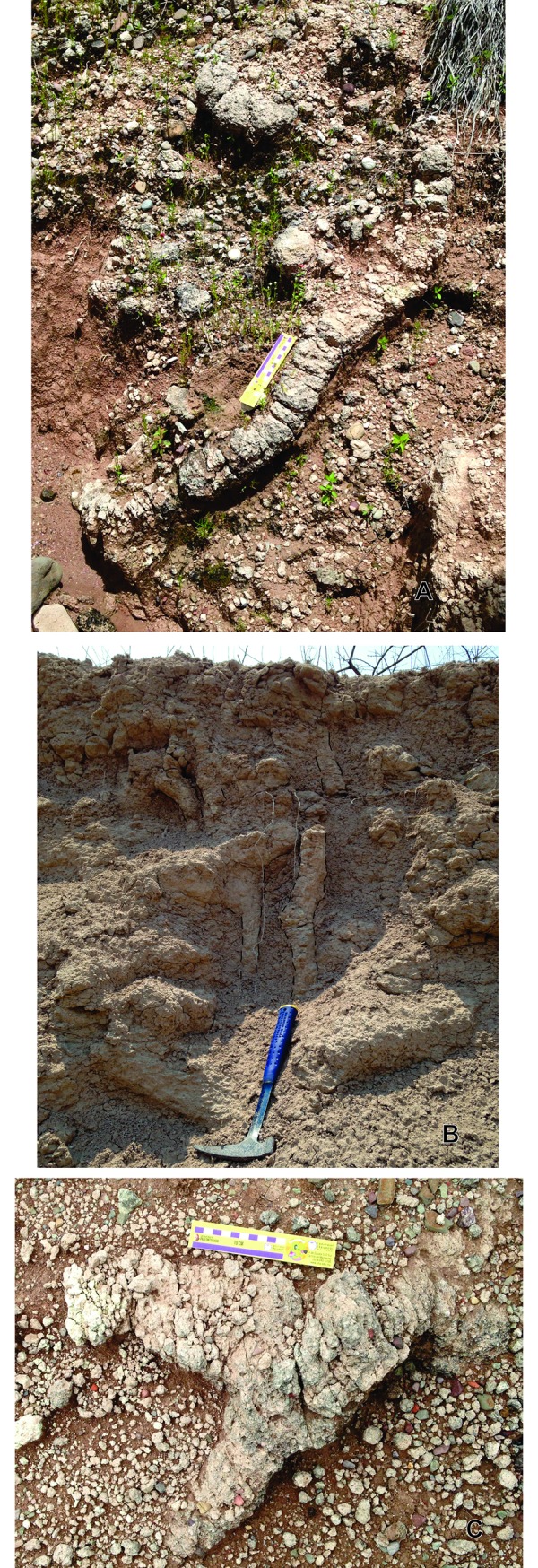
General morphology of *Y*. *iniyooensis* vertical to sub-vertical tunnels. (A) Sinuous segment of a tunnel, probably descending from a secondary chamber. Divisions of the rule are in centimeters; total length of the rule = 17 cm. (B) Vertical and sub-vertical straight segments of tunnels delineated in black. Scale = 34.3 cm. (C) Segment of a bifurcated tunnel. Divisions of the rule are in centimeters; total length of the rule = 17 cm. All the tunnels are *in situ*, in “middle beds” strata.

**Description.** Main chambers are roughly ellipsoidal and flattened in shape, but some of them are deformed by carbonate deposition and weathering, therefore looking more distorted. The best exposed main chambers measure 35–90 cm wide and 47–100 cm long (n = 5), with a height of 18–90 cm (n = 2) (S2 File) (Fig 4B and 4D; delineated in red). They are located near the paleosol top. The entrance tunnel was not preserved. Main chambers show lateral and horizontal burrows, as well as vertical or sub-vertical shafts radiating from the lower part of the chamber (Fig 4B–4D; delineated in blue). Horizontal to sub-horizontal tunnels range from almost straight to sinuous, or they show a “C-” or “H-” path (Fig 7). The longest fragment of a horizontal tunnel measured *in situ* is 135 cm. The number of vertical to sub-vertical, radiating burrows are variable, but mostly they are five or six. Vertical and sub-vertical shafts are straight, sinuous, curved or showing successive arches resembling a roughly helical design; they are simple or bifurcated (Fig 8). They extend 8 to 10 m below the chambers. Some vertical shafts (n = 9) are completely straight and almost circular in cross section; minor diameter ranges from 5.1 to 8.7 cm; whereas major diameter ranges from 5.5 to 8.9 cm (S3 File). Secondary chambers are smaller than main chambers and located at the end of shafts or are lateral to them. They are 20–32 cm wide, 24–47 cm long height (n = 11) (Fig 4B–4D; delineated in yellow). Cross-sections of horizontal, sub-vertical and some vertical burrows are elliptical (Fig 5). The width of burrows ranges from 6.5 to 14.4 cm, whereas height ranges from 5.4 to 14 cm (n = 71) (S3 File).

**Fig 9:**
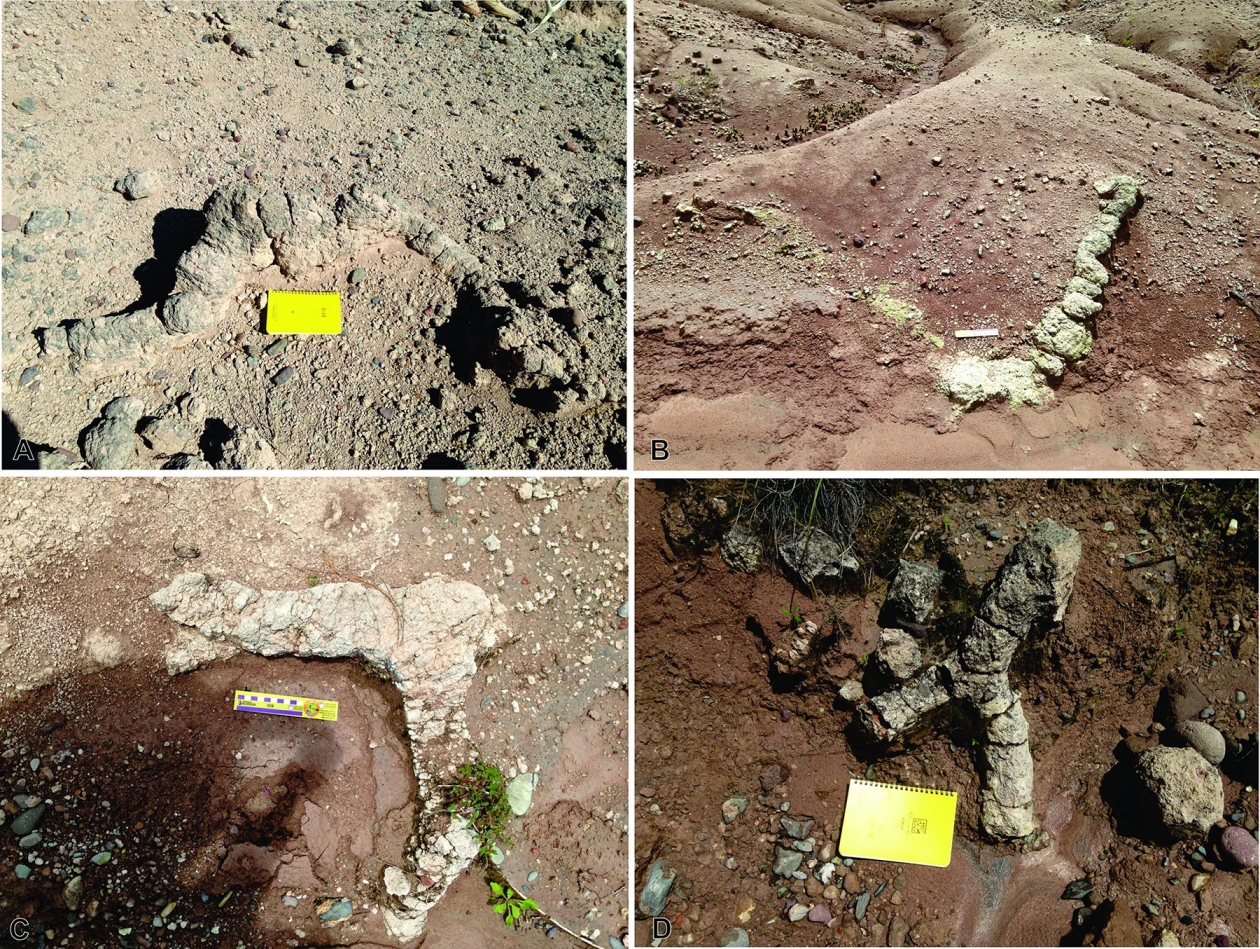
Burrows showing the characteristic arrangement in packets of the sediment fillings, indicating of active burrowing. **(A)** Secondary chamber and segments of an almost straight tunnel. Length of the scale = 18 cm. (b) Isolated segment of sinuous tunnel and remains of a secondary chamber. Length of the scale = 17cm. (c) Remains of a horizontal tunnel, originally bifurcated, (D) Sub-horizontal segment of a tunnel, bifurcated. All the specimens are *in situ* from “middle beds” strata.

Some fillings are arranged in clumps, which were more observable in weathered specimens (Fig 9). Packets were 1.2–10 cm long (n = 44) (S4 File). They were found in the “lower beds” and in some paleosols of the “middle beds.”

Several casts with fine- to medium-sized sediment fillings show paired grooves on the external surface (0.29–1.11 cm wide, 1.25–8.5 cm long; n = 33) (Fig 6) (S5 File). Most of these traces are oriented with their long axes parallel to the long axis of the burrow, but others have an almost perpendicular orientation (Fig 6B). They are distributed mainly in the ceiling and the lateral sides of the tunnels. Bioglyphs were not detected in the walls of chambers. Casts with bioglyphs are especially abundant in the upper horizons of the “upper beds”.

**Fig 10:**
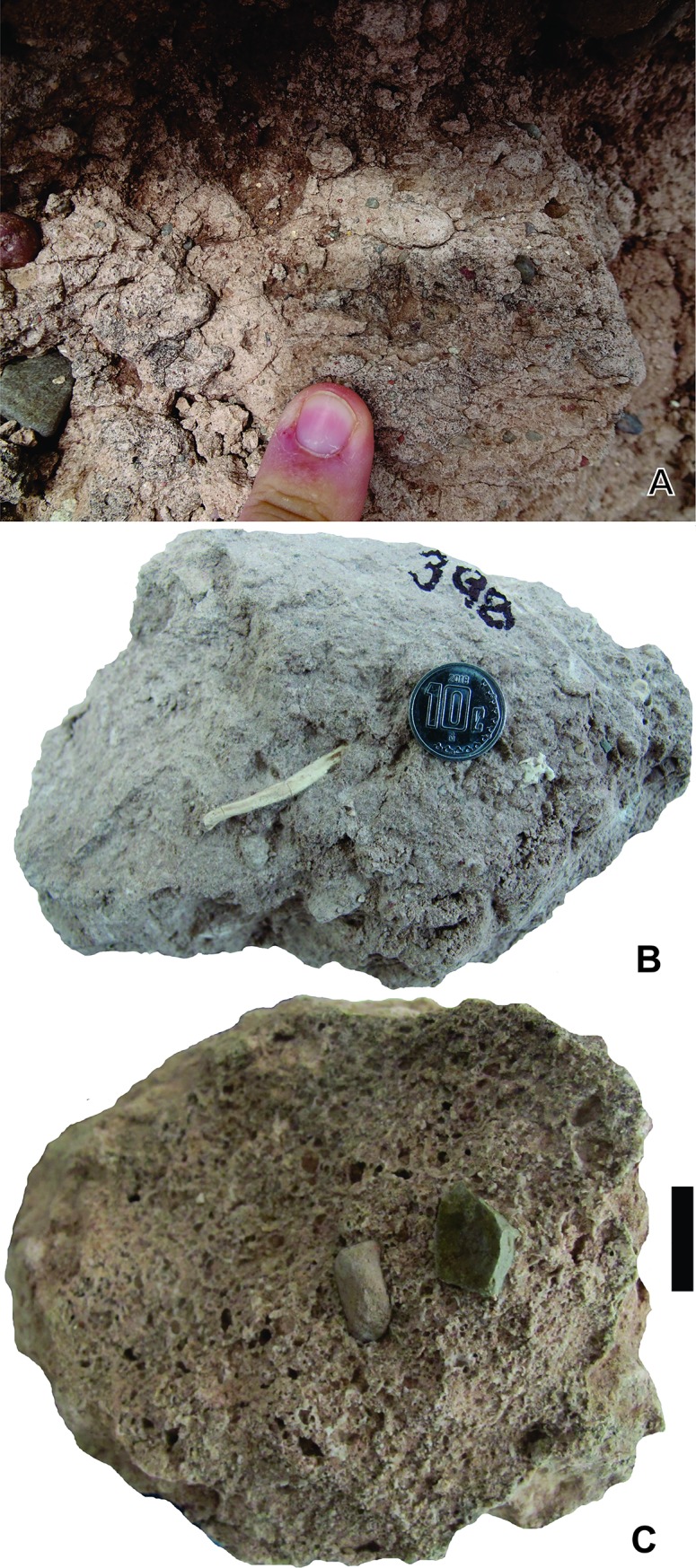
Remains inside the fillings of *Y*. *iniyooensis*. (A) A cast of *Fictovichnus gobiensis* inside the filling of a tunnel. (B) Remains of poscraneal remains of *G*. *veloxikua* inside the filling of paratype UMPLIC-398. Diameter of the coin = 4 mm. (C) Lithics inside the filling of paratype UMPLIC-393. Length of the scale = 20 mm.

Some isolated horizontal burrows have coarser sediments (pebbles and cobble-sized grains) inside the filling (Fig 10C).

## Discussion

### Potential producers of *Yaviichnus iniyooensis*

Iniyoo Local Fauna contains only four fossorial representatives: the squamate *Rhineura* (Amphisbaenidae) and three taxa of rodents (*Gregorymys veloxikua*, *Gregorymys* sp. and Florentiamyidae indet.) [5,6]. *Yaviichnus* is composed of chambers and burrow systems different and much larger from those produced by amphisbaenians. Burrows of amphisbaenians (like *Rhineura*) consist of complex, interconnected networks, with multiple branches per junction, composed of cylindrical, sinuous or straight tunnels. The surface morphology consists of triangular impressions on the top and sides of tunnels [11].

It is probable that the producer of *Y*. *iniyooensis* was a rodent, not just because the only other fossorial components of the Iniyoo Local Fauna were rodents, but also because of the presence of paired traces in the walls of the burrows, which are usually produced by rodent incisors from gnawing and breaking the soil [12,13]. The presence of incisor traces may have two explanations: producers were probably juvenile organisms that preferred to use their incisors instead of their weaker forelimbs [14]; or soil was so hard that the producer used their incisors to loosen the soil more effectively [15]. The former hypothesis is unlikely considering that no forelimb traces were found in any burrows, where adults had to have been present as well.

Compared to other fossilized chamber and tunnel systems probably produced by Geomyidae, *Yaviichnus* is different with its unique arrangement of chambers, associated tunnels, and bioglyphs (Table 1).

**Table 1:** Main features of fossil burrow systems probably produced by geomyidae.

	Burrow system morphology	Cross-sectional diameter of tunnel	Bioglyphs	Chamber	Producer
*Alezichnos trogodont* [16]	Tubular and sinuous tunnels, varying directionality, and irregular orientation pattern. Some are weakly helical in the vertical plane.	5.6–6.7 cm	Claw marks and incisor grooves. Abundant, groove-like, flat-edged mark. Narrower ridges, grooves situated end to end. Abundant on the probable ceiling of the runnel.	Bilobate.	Possibly *Gregorymys*
Unnamed burrows [13]	Five main types of burrow system, varying in the disposition of chambers and tunnels. Tunnels subdivided in horizontal and near-horizontal, vertical and near-vertical shafts, and diagonal tunnels trending at angles between 20° to 70° from the horizontal.	8.98 cm in average	Paired striations in the walls, with uniform depths and widths, interpreted as the results of tooth digging.	Two types: type 1 are highly elongate and tunnel-like features, slightly greater in diameter than the tunnels *per se* that enter them; type 2 are irregular features with roughly circular, oval, or squarish vertical sections.	Two probable producers were considered: Geomyidae or marmotine ground squirrels.
*Yaviichnus iniyooensis*	Main large chambers near the top of paleosol, horizontal, subhorizontal, vertical and subvertical shafts with different morphologies and orientations radiate from main chamber. Small, deeper chambers may be present.	Vertical shafts almost circular with a major diameter range of 5.5–8.9 cm, and minor diameter range of 5.1–8.7 cm.	Paired grooves on the external surface, interpreted as incisor traces, distributed mainly in the ceiling and lateral sides of the tunnels.	Two types: a main and large chamber, roughly ellipsoidal and flattened in shape; secondary chambers smaller than the main chambers, located at the end of the shafts or are lateral to them.	Possibly *Gregorymys veloxikua* and *Gregorymys* sp.
		Elliptical shafts with a width of 6.5–14.4 cm, and width of 5.4–14 cm.			

Only two fossil rodent burrow systems have been associated directly to geoymids: *Alezichnos* and *Daemonelix*. *Alezichnos trogodont* has been attributed to geomyids [16]. *Alezichnos* consists of primary tunnels, which occasionally branch into secondary ones. It lacks main and secondary chambers, as well as the diversity of tunnel morphologies and orientations observed in *Yaviichnus* [16,17]. Morphology also differs in *Alezichnos* and *Yaviichnus*. *A*. *trogodont* has sinuous, tubular morphology with varying directionality and bilobated chamber; *Yaviichnus* is a system composed by two types of chambers, and horizontal and vertical tunnels. *Y*. *iniyooensis* burrows have paired grooves related exclusively with incisors; *A*. *trogodont* has small scratches and grooves on the surface of the ceiling and upper halves of burrows, produced by a combination of incisors and claws; the entire surface of the chamber is covered with regularly spaced claw marks [16]; this combination was not observed in *Yaviichnus*, and as will be discussed further, bioglyphs are probably related to soil type. The other fossil burrow associated with *Gregorymys* is *Daemonelix*, due to the presence of remains of this geomyid inside these helical burrows that are attributed to *Paleocastor* [18]. Architecture of *Daemonelix* is clearly different from *Yaviichnus*, since the first is a vertical, helical shaft with an inclined chamber at the base [16, 18].

Two main arguments could be considered to argue that *Gregorymys* is not the potential producer of *Yaviichnus*. The complexity of *Y*. *iniyooensis* is not similar to any extant geomyid burrow systems. They consist of a less complex architecture: a main burrow, generally 10–46 cm below and parallel to the ground surface, with a variable number of lateral burrows branching from the main one; there are also deeper branches that are used as nests and food stores [19,20]. These simple burrow systems of geomyids are related to the reported solitary habits of all known species [21]. *Y*. *iniyooensis* differs notably from this pattern. However, neither does the configuration of modern system match with other fossil burrows attributed to *Gregorymys* spp. [13, 16].

The presence of remains of *Gregorymys* inside *Y*. *iniyooensis* might be also explained by passive transport: 30% of the cranial and postcranial remains of *Gregorymys* collected in Yolomecatl were recovered from casts of *Y*. *iniyooensis*, whereas the remaining 70% was collected in the rock matrix and is apparently not associated with the burrows. An alternative hypothesis to explain the low percentage of remains inside the fills is that *Gregorymys* were secondary occupants of the burrows, as is interpreted in other burrows produced by large mammals [18].

We consider *Gregorymys* spp. as the most probable producer of *Yaviichnus* in Yolomecatl localities by several reasons. *Gregorymys veloxikua* and *G*. sp. are the only taxa of fossorial rodents in Iniyoo Local Fauna, where their remains are as abundant as the burrows. No other fossorial vertebrate species was identified in the Yolomecatl outcrops to be considered as the main inhabitant and excavator of the burrow systems [5,6, 22]. Some remains were found in burrows with active fillings, resulted from the behavior of backfilling; active fillings could be identified because they have the same lithology of the rock matrix, and in some cases are structureless. Low percentage of remains of potential producers inside the fills could be explained because animals could scape from floods, before they entered into the burrows.

A critical piece of evidence to relate *Y*. *inyooensis* to *Gregorymys* is that the paired traces on the external surface of casts match with the width of the incisors of this geomyid (Fig 6). These bioglyphs indicate chisel-tooth digging. Even though the primary digging mode among geomyids is scratch digging, to some extent they also use their procumbent incisors to break up soil as a secondary digging mode [20], especially in hard soils. Florentiamyidae individuals can be disregarded as producers because their incisors are much smaller and thinner (approximately 40–50%) than the traces recorded in the burrows.

So, evidence reveals that Oligocene Geomyidae in southern Mexico produced different burrow systems that appear to be more complex than any other extant or fossil representatives of this family. The reasons for this behavior are discussed in the following sections.

### Burrow system function related to paleoenvironment

The functions of the different components of the system represented by *Y*. *iniyooensis* may be interpreted according to the knowledge of similar morphologies for burrows of extant species.

In recent geomyids, chambers could be used as nests, latrines or food storages [21]. In *Y*. *inyooensis* the original fillings of chambers were replaced, so it was not possible to distinguish between those chamber types. Large chambers in other rodent burrows may have a nesting and/or a thermoregulatory function [23, and references therein], or can be used as latrines or for food storage [21, 24 and references therein]. Burrows tend to become more complex in mammals whose entire existence occurs underground [25] because they display different functions, such as shelter, protection from external conditions and provision for the development of juveniles.

*Y*. *iniyooensis* has vertical and horizontal burrows. Descending sub-vertical and inclined shafts (Fig 8B) could be used for thermoregulation, since it is observed that burrowing mammals dig deeper when environmental conditions become more severe by an increase in temperature and/or lack of humidity [3]. Completely straight vertical tunnels (Fig 8B) could be also used as drainage canalization [13]. Horizontal burrows (Fig 7A) could be used when searching for food resources underground. In modern systems they may run across different vegetated areas and through soils of different types [3]. It has been observed that fossorial rodents (like *Ctenomys)* construct longer and more complex burrow systems in an environment with lower food cover and availability [26].

Accordingly, large chambers, sub-vertical and long horizontal burrows in the same system could be convergent evidence of non-optimal conditions for life on the surface, which can be tested with other evidence recovered from the Yolomecatl strata.

**Fig 11:**
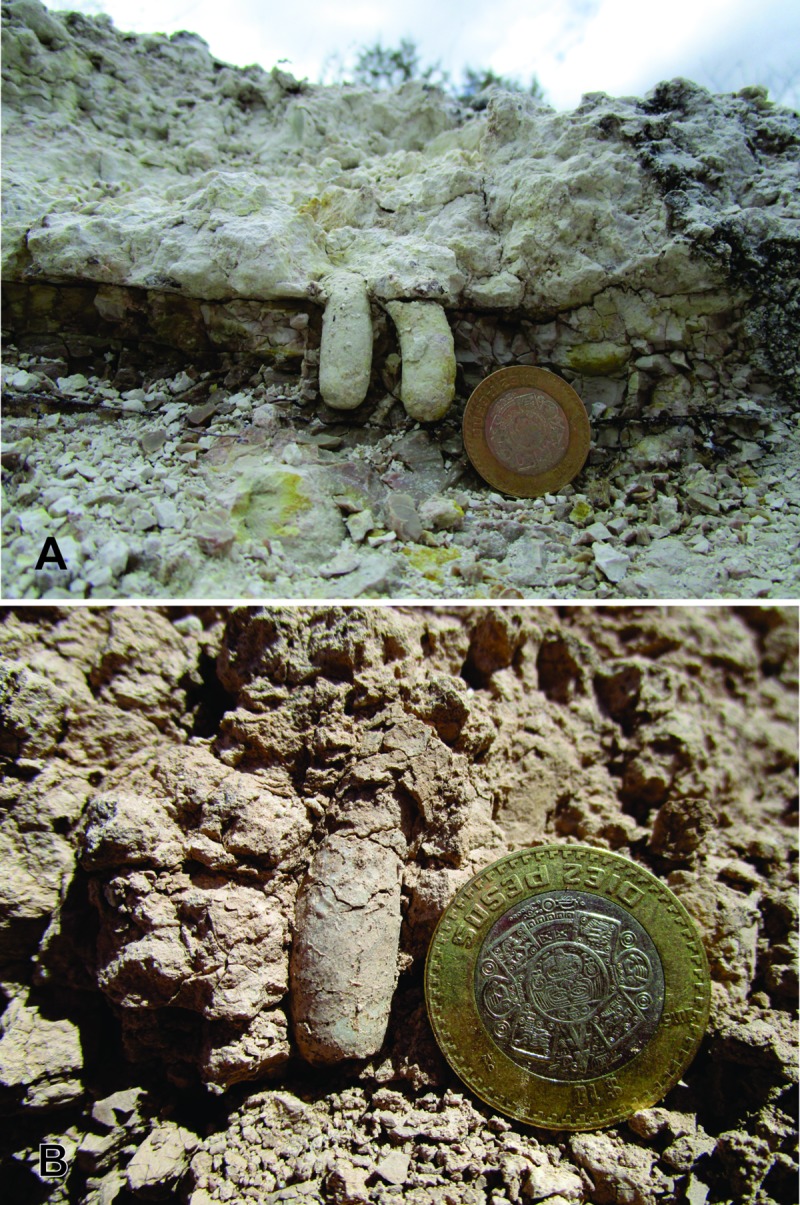
Insect trace fossils found in the same stratigraphic levels as *Y*. *iniyooensis*. (A) Specimen of *Cellicalichnus*? isp. from the “lower beds”; **(B)** *Celliforma* isp. from the “middle beds”. Diameter of the coin in both figures = 28 mm.

Trace fossils produced by insects, such as *F*. *gobiensis*, *Teisseirei barattinia* and *Celliforma* ispp (Fig 11) reported previously [7], are representative of the *Celliforma* Ichnofacies, indicative of scrub and woodland of arid to semiarid environments, or of palustrine vegetation or bare soils due to frequent flooding [27]. The paleolandscape was inferred as a scrubland or a woodland with a low vegetation cover [7]. This was recently confirmed by the presence of interbedded calcrete layers among the sequence and by dolomite, zeolite and attapulgite minerals, which are also indicative of aridity [10].

It is well documented that a major climatic change occurred during the transition from the Eocene to the Oligocene. In response to this process, global aridification and the emergence of an open-habitat biota occurred at the beginning of the mid- to late Cenozoic [15, and the references therein]. Climatic changes corresponded to a period of extensive diversification of subterranean taxa, and their tendency to occur in open, arid or semiarid habitats. Fossil plants and pollen records show that a development and expansion of xeric vegetation in Mexico occured during the Oligocene (33–23 ma), when lowland forests and chaparrals were established in the central part of the country [28].

The presence of exclusively incisor traces in the walls of *Y*. *iniyooensis* could be also related to soil characteristics in arid or semiarid landscapes. Soil conditions (hardness, degree of compactation or dryness) have a significant effect on whether a digger adopts the tooth- or claw-digging strategy or combines both [17]. There is a tendency in extant rodents such as *Thomomys bursarius* and *Cynomys leucurus* to use incisor-digging in particularly harsh and dry conditions [17, and the references therein]. The chisel-tooth digging style is common in other rodents, like mole-rats, suggesting that incisors have been used as the main tools to enable exploitation of hard soils [29–31]. The presence of incisor traces in the walls indicate that the soil in Yolomecatl was compact and dry; these conditions are related to the proposed environment.

Lithic fillings suggest that flooding events or gravity probably filled these burrows. Flooding events were related with intermittent currents of high energy recorded in the area due to seasonality in the area, related with the global tendency of climatic change during Oligocene [10].

### Burrow system function related to sociality

There are socioecological hypotheses to explain the evolution of sociality among rodents. There are various causes and factors proposed to promote cooperative behaviors among individuals such as predation pressure, distribution of food resources and environmental and climatic conditions [32, and the references therein]. One of the most cited proposals to explain sociality in rodents is the conceptual model of the Aridity-Food-Distribution Hypothesis (AFDH). This hypothesis postulates that the abiotic factors that cause patchy distribution of food resources promote social interactions between individuals, and therefore the population’s survival [33–37]. The energetic cost of burrowing through hard soil to locate patchily distributed but locally abundant food resources is the primary selective factor favoring group-living; by living together and working cooperatively to excavate tunnels, the animals can locate enough food resources to survive [32]. Subterranean foragers might be expected to modify their burrow architecture in different habitats, coinciding with different food resource idiosyncrasies [34]. Burrowing animals are limited to habitats where burrow excavation is energetically efficient, which is determined in part by the nature of the soil and the associated vegetation, among other factors [35].

In low productivity areas, more extensive exploration might be required to locate resources [36]. The relative costs of social versus solitary diggings allow the presumption that complex structures were produced by more than one organism [37]. The high branching of a burrow system is also expected to increase with the number of inhabitants [36, 38]. The complexity of the burrow system represented by *Y*. *inyooensis*, composed of interconnected large and small chambers at different depths and vertical to horizontal burrows showing different morphologies and functions, plus the extension of these burrow systems, may suggest that the systems were constructed by more than one individual. A hypothesis for the construction of these burrows is that they were produced by more than one individual, in order to diminish burrowing costs in an environment with relatively scarce food resources. Arid or semiarid climate in Yolomecatl, evinced by paleosols, minerals and ichnofacies, probably produced a patchy and scarce distribution of vegetation sources for fossorial organisms; also, periodical flooding events could contribute to a patchy distribution of resources in the area.

### Non-solitary oligocene geomyids?

Rodentia encompasses a vast array of social systems, which range from short-term seasonal aggregations to long-life social groups [32, 39]. If a non-solitary *Gregorymys* could be considered as the producer of *Yaviichnus*, it is implied that the Oligocene species had different habits from extant geomyids, which are reported as solitary during their entire life cycle, even under arid climates [40]. However, it is possible to find solitary and social species within the same taxon in subterranean rodents, e.g. *Ctenomys sociabilis* [41,42] and *Ctenomys haigi* [41]. Recent and relevant evidence of ecological genomics shows how genetics influence the evolution of complex behavioral differences of modern rodents in nature. Genetic changes contribute to the evolution of different architectures of burrows, even between closely related species (like *Peromyscus polionotus* and *P*. *maniculatus*) [43,44]. Novel observations suggest that social organization in rodents is influenced by changing environmental conditions [45,46]. Our hypothesis is that *Gregorymys* individuals in Yolomecatl could have some degree of social organization in environments triggered by aridity conditions.

The question of which degree of gregariousness was showed by Yolomecatl geomyids remains open, but it is more probable that association of individuals was short and associated with cooperative behavior under certain climatic conditions, as it is observed with extant bathyergids in arid landscapes [47, and the references therein]. The fossil record offers an invaluable source of evidence of novel and unobserved combinations of behavioral characteristics never observed in living species [48]. *Yavichnus inyooensis* could provide a new window to explore the evolution of social life in rodents during the Oligocene of southern Mexico.

The composition and importance of the burrowing herbivore guild changed during the Cenozoic. Convergent evolution of subterranean mammals began across the planet during the global climatic transition from the middle Eocene to the early Oligocene (40–30 Ma) [2]. As environments became more open in the Cenozoic, small herbivorous mammals would opportunistically exploit small patches of open habitats and show rapid adaptations for life within new habitat types [49] and subterranean life [2]. During the Arikareean (30–18.5 Ma) entoptychine geomyids were an important component of North American faunas [49]. The extension of burrowed paleosols and complexity of the burrows by the dominant geomyids in Yolomecatl strata support both assertions.

## Conclusions

1) *Yaviichnus inyooensis* is a new ichnotaxon for complex burrow systems composed of interconnected large and small chambers at different depths, as well as vertical to horizontal burrows, showing different morphologies and functions.

2) A species of *Gregorymys* would be the most probable producer based on its fossorial habits, the presence of its remains inside the burrows and the paired grooves in the walls, which are compatible with rodent incisors.

3) The complexity of these burrows and underground life would have been triggered by semiarid to arid conditions shown by independent evidence such as paleosols, minerals and ichnofacies.

4) The morphological complexity of burrows could be related with the action of more than one individual, indicating that the Oligocene *Gregorymys* of southern Mexico shows some degree of gregariousness influenced by environmental conditions.

## Supporting information

S1 File Supporting information on the U-Pb detrital zircon geochronology. (DOCX)

S2 File Measurements of chambers of *Yaviichnus iniyooensis* specimens. (XLSX)

S3 File Measurements of diameter of tunnel of *Yaviichnus iniyooensis* specimens. (XLSX)

S4 File Measurements of width of packets of sediment of *Yaviichnus iniyooensis* specimens. (XLSX)

S5 File Measurements of length and width of bioglyphs of *Yaviichnus iniyooensis* specimens. (XLSX)
