# Supplementary material for: Burrow systems evince non-solitary geomyid rodents from the Paleogene of southern Mexico
Source: PLoS One. 2020 Mar 12;15(3):e0230040. doi: 10.1371/journal.pone.0230040 (PMC7067467; doi:10.1371/journal.pone.0230040)
Supplement: S1 File — (DOCX) [file pone.0230040.s001.docx]

**Burrow systems evinced probable sociality in geomyid rodents from the Paleogene of southern Mexico**

Rosalía Guerrero-Arenas, Eduardo Jiménez-Hidalgo and Jorge Fernando Genise

**Supplementary materials**

**1.- U-Pb detrital zircon geochronology**

A sandstone bed within the Yolomécatl sequence was analyzed to estimate its maximum depositional age. This sample comes from the immediately overlying ichnofossiliferous bed (Fig 2).

Sample preparation followed standard techniques [1]. The analyses were carried out at Laboratorio de Estudios Isotópicos, Centro de Geociencias, Universidad Nacional Autónoma de México, employing a Thermo iCapQc ICPMS coupled to a Resonetics, Resolution M050 excimer laser workstation.

           Concordant U-Pb data were yielded by 26 zircons (Table 1). To estimate their maximum depositional ages, the zircon data of each sample were log-transformed, analyzed with the minimum Mixture Model of DensityPlotter software and plotted with the kernel density Estimator of the same software [2].

**References**

1. Solari LA, de León RT, Hernández-Pineda G, Solé J, Solís-Pichardo G, Hernández-Treviño T. Tectonic significance of Cretaceous–Tertiary magmatic and structural evolution of the northern margin of the Xolapa Complex, Tierra Colorada area, southern Mexico. Geol Soc Am Bull. 2007;119:1265-1279.

2. Vermeesch P. On the visualization of detrital age distributions. Chem Geol. 2012;312-313:190-194.

Table 1. Concordant data of detrital zircons from Santiago Yolomécatl , Oaxaca, southern Mexico

| 1: U and Th concentrations are calculated employing an external standard zircon as in Paton et al., 2010, Geochemistry, Geophysics, Geosystems. | | | | | | | | | | | |
| --- | --- | --- | --- | --- | --- | --- | --- | --- | --- | --- | --- |
| 2: 2 sigma uncertainties propagated according to Paton et al., 2010, Geochemistry, Geophysics, Geosystems | | | | | | | |  |  |  |  |
| ^207^Pb/^206^Pb ratios, ages and errors are calculated according to Petrus and Kamber, 2012, Geostandards Geoanalytical Research | | | | | | | | | |  |  |
| Analyzed spots were 23 micrometers, using an analytical protocol modified from Solari et al., 2010, Geostandards Geoanalytical Research. | | | | | | | | | | |  |
| Data measured employing a Thermo iCapQc ICPMS coupled to a Resonetics, Resolution M050 excimer laser workstation. | | | | | | | | |  |  |  |
|  |  |  |  |  |  |  |  |  |  |  |  |
